# Patterns of crystallin distribution in porcine eye lenses

**Published:** 2008-07-04

**Authors:** J. Keenan, D.F. Orr, B.K. Pierscionek

**Affiliations:** School of Biomedical Sciences, University of Ulster, Coleraine, UK

## Abstract

**Purpose:**

To measure the protein distribution patterns in single young porcine lenses.

**Methods:**

Twenty fresh porcine lenses from 5 to 6 months old animals were fractionated into 8–10 concentric fractions by controlled dissolution in phosphate buffer. Proportions of soluble and insoluble protein were determined by Bradford assay. Water-soluble proteins in all layers were separated into HMW, MMW, and LMW fractions by size-exclusion HPLC and constituents of each class further characterized by SDS gel electrophoresis, as were the water-insoluble proteins. Size-exclusion fractions were further separated by reverse-phase HPLC and the molecular masses of each peak determined by MALDI-TOF mass spectrometry.

**Results:**

The major soluble proteins in the porcine lens are β-crystallins. They comprise around 45% of the total protein in the outer lens decreasing gradually to 35% in the central region. Soluble α-crystallins vary from 35% to 22% from outer to inner lens. The proportion of soluble γ-crystallin levels, substantially lower than that of the other protein classes, increases gradually with progression into the lens center. Insoluble protein levels also increase from outer to inner lens layers.

**Conclusions:**

In the young porcine lens, there is relative constancy in the levels of all three crystallin classes in the outer lens with α- and β-crystallins representing the predominant protein classes. The increase in γ-crystallin in the inner lens may contribute to the refractive index gradient.

## Introduction

The lens of the eye lens is a highly specialized, avascular organ that is functionally required to provide refractive power and maintain its transparency. The lens remains transparent because of an organized arrangement of its structural proteins; the crystallins [1,2]. While it is considered that short range order is sufficient for maintenance of transparency [3] and there is some experimental support for this in the fresh lens [4], evidence of long range order in the protein arrangement in dehydrated lenses has been found [5]. The structural intricacy of proteins, coupled with the fact that, in mammalian lenses, there are three broad classes of crystallins [6], with varying distributions across the tissue [7,8], renders the structure/function relationship in the lens one with varying levels of complexity.

An important feature of the lens is that it continually grows throughout life and accumulates cells in its outer layer without any protein turnover. Because of this unique pattern of cell accrual, it is an ideal tissue to study from a growth and aging perspective. Changes in lenticular protein distribution are a result of changing patterns of synthesis. The effect of post-translational modifications such as deamidation, phosphorylation, racemization, isomerization, oxidation, and backbone cleavage [9,10] are superimposed onto growth patterns.

There is no universally adopted method for extraction of lens proteins; previous studies have largely examined pooled whole lenses or separated nuclear and cortical fragments. The shortcoming with this approach is that patterns of growth are masked and age-related changes averaged over the whole lens, or large sections of it.

A better approach is to study concentric lens layers following the growth pattern of the lens [11-17]. Separating the lens into several concentric layers by controlled dissolution overcomes many of the disadvantages inherent in alternative techniques [15-17]. This methodology has previously been applied to bovine, human, fish, and porcine lenses to examine protein distribution patterns [14-17]. Since protein concentration is related to refractive index [18], it has been suggested that the protein distribution patterns may have some bearing on the refractive index gradient in the lens [8]. Knowledge of how the different protein classes vary across the lens with cell growth and how these changes impact on the optics of the lens may provide important insight into the structure/function relationship.

This study has investigated the distribution patterns and characterization of proteins in porcine lenses, a species in which comparatively little data on the structure/function relationship exists but one which has been suggested as a suitable model for the human lens [17]. This suggestion is supported by findings showing close sequence homology between human and porcine αB-crystallins [19] and by the results of immunochemical studies that report highly significant antigenic similarity between human and porcine crystallins [20]. In addition, rheological investigations indicate that the complex shear modulus of the porcine lens resembles that of its young human counterpart [21]. In this study, young porcine lenses were used to determine patterns of growth without the confounding factor of aging.

## Methods

Twenty porcine (*Sus scrofa domestica*) eyes were obtained from an abattoir in Co. Antrim, Northern Ireland. All eyes were from pigs of the same age (5 - 6 months), similar genetic pool, and single breed of pig (25% *large white*; 75% *landrace*) reared under uniform conditions. Within eight hours of slaughter, lenses were removed from the eye, weighed, and either frozen at −20 °C or used immediately for experimentation. All experiments were performed at room temperature unless otherwise stated.

### Lens protein fractionation

Lenses were decapsulated and successive concentric fractions isolated through step-wise dissolution by gentle agitation in 1 ml of phosphate buffer (pH 7.3) [15,16]. Using this method, 6–10 extracts were obtained for each lens. Each extract was then centrifuged at 11,000x g for 20 min at 4 °C to separate water-soluble protein from water- and urea-insoluble fragments. Protein contents (soluble and insoluble fractions) were determined by the Bradford assay [22] using BSA as standard. Measurements of each sample were made in triplicate. Water-soluble and -insoluble protein contents were expressed as a proportion of total protein in each lens layer.

### Size-exclusion chromatography

Soluble proteins from each lens were separated into high, medium, and low molecular weight fractions by size-exclusion chromatography using a Waters Biosuite 250 5 μm HR SEC column (7.8x300 mm) attached to a Waters HPLC system (Waters, Milford, MA) at a flow rate of 1 ml/min and detection at 280 nm. The column was calibrated using gel filtration standards. At least two separations were performed on each sample and fractions were collected from each peak. Areas under each peak were calculated using Waters EmPower software and protein proportions determined from these areas corrected for respective extinction coefficients (0.7, 2.1, and 2.1 ml.mg^−1^.cm^−1^ [17]) after protein characterization.

### SDS Gel electrophoresis

Samples from each chromatographic peak were dried at room temperature using a Concentrator 5301 (Eppendorf, Hamburg, Germany). Each preparation was then subjected to SDS gel electrophoresis by the Laemmli [23] method using 4%–12% NuPAGE Novex Bis-Tris Pre-cast Gels (10x10 cm) following the NuPAGE Electrophoresis system protocol (NuPAGE, Invitrogen, Carlsbad, CA). Insoluble protein fractions, dissolved in RIPA buffer, were also electrophoretically analyzed. Gels were stained with Coomassie blue (SimplyBlue Safestain; Invitrogen).

### Reverse Phase HPLC

Selected samples of each size-exclusion peak (HMW, MMW and LMW peaks) were individually subjected to RP-HPLC fractionation using a 4.6x150 mm, 5 μm analytical Phenomenex C-8(2) column (Hichrom, Reading, Berks, UK) eluted at a flow rate of 2 ml/min with a gradient of TFA/water (0.1:99.9, v/v) to TFA/water/acetonitrile (0.1:19.9:80.0, v/v/v) in 80 min using a Waters separation system. The column effluent was monitored simultaneously at 214 nm and 280 nm and fractions (1 ml) were collected and numbered at 1 min intervals.

### Molecular Mass Determination

MALDI-MS was performed on all peaks using an Applied Biosystems Voyager instrument with delayed extraction and autosampler (Foster City, CA). Samples were prepared by mixing a 1 μl aliquot with 1 μl of matrix solution. The matrix solution was made by saturating a water–acetonitrile (50:50), 0.05% TFA solution with sinapinic acid. Samples of 1 μl were spotted into wells of the MALDI sample plate and allowed to air-dry before being placed in the mass spectrometer. All peptides were analyzed in the linear, positive ion mode by delayed extraction using an accelerating voltage of 20 kV, unless otherwise noted. The limit for detection using this system was 10 fmol. External calibration was achieved using a standard “calibration 2” mixture from PE Biosystems.

## Results

**Figure 1:**
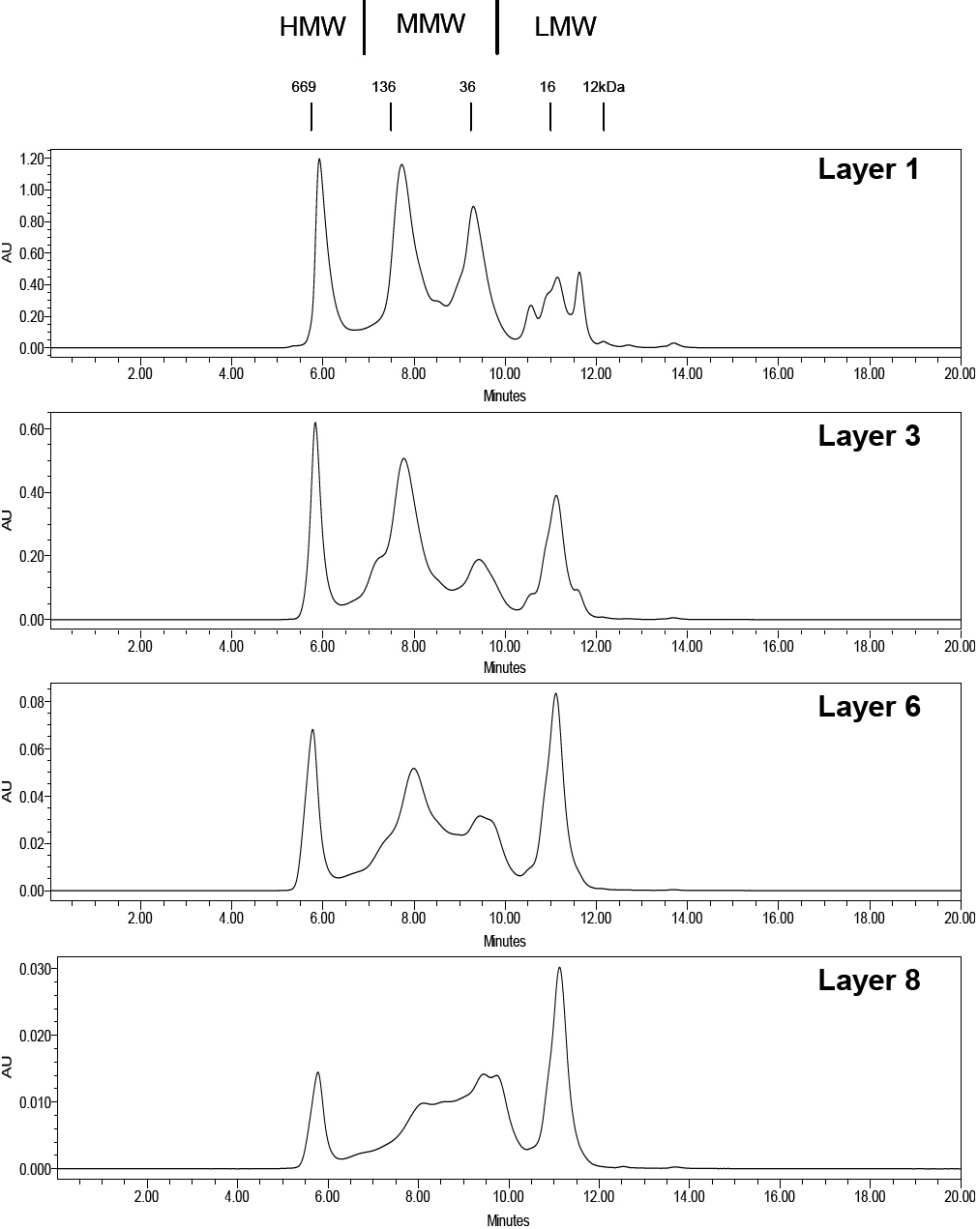
Typical size-exclusion HPLC elution profiles of the water-soluble proteins from selected layers of porcine lens.  The layers are numbered consecutively from the lens periphery into the center.  Numbers above the chromatogram indicate positions at which various molecular weight standards eluted.  From lens periphery to center, HPLC profiles show a decrease in HMW proteins, an increase in LMW proteins and a change in proportion of the MMW proteins.

Lens wet weights ranged from 0.345 to 0.493 g with total protein contents representing between 33.77-35.8% of the wet weight. Representative chromatographs obtained from selected layers are shown in Figure 1. With progression from the periphery to center, there is an almost constant proportion of the HMW peak with a slight decrease in central layers. The MMW peaks show two major species, with a predominance of the larger of the MMW species in the outer layers and a higher proportion of the lower of the MMW species in the central layers. The MMW proteins from these layers (laid down pre-natally) show poorer separation compared to the separation obtained from newly synthesized outer layers, a finding which has previously been observed with porcine lenses [17]. The LMW proteins appear as three peaks in the outer layers, merging into a single peak in the central layers.

**Figure 2:**
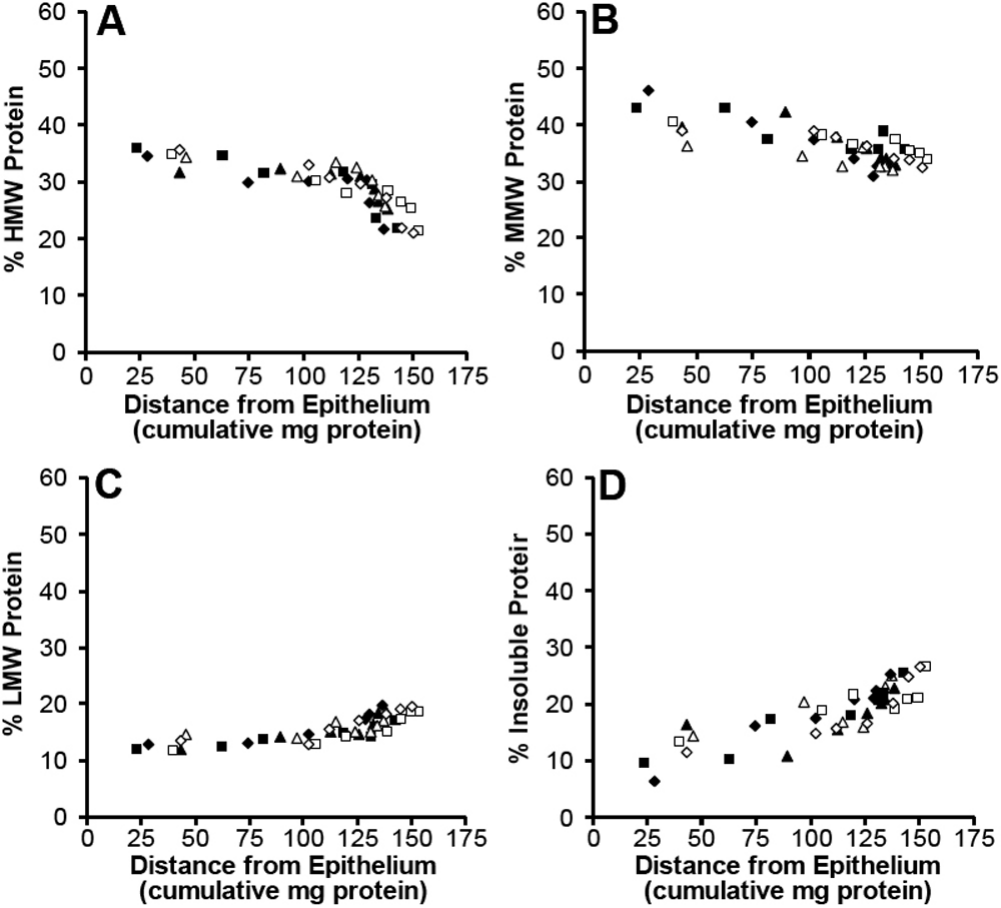
Protein distribution patterns in 6 representative porcine lenses.  The proportions of HMW protein (**A**); MMW proteins (**B**); LMW proteins (**C**) and insoluble protein (**D**) are plotted as a function of the cumulative amount of protein from the lens periphery to centre.  With progression towards the lens center there is a decrease in the HMW and MMW proteins and a concomitant increase in LMW and insoluble proteins.

The proportions of each class of proteins under the peaks are plotted in Figure 2 against the cumulative weight of total protein from lens periphery to center. Proportions were obtained by calculating the area under each peak and normalizing the data. The HMW proteins represent around 35% of the total soluble protein in outer lens layers decreasing to about 22% in the central layers (Figure 2A). The total of the MMW protein species varies across the lens changing in proportion from around 45% of the total soluble protein in the outer layers to around 35% in the central layers (Figure 2B). The LMW proteins increase from around 12% of the total soluble protein in the outer layers to about 18% in the central region (Figure 2C). There is an increase in insoluble proteins from around 8% in the outer layers to around 25% in the inner layers.

**Figure 3:**
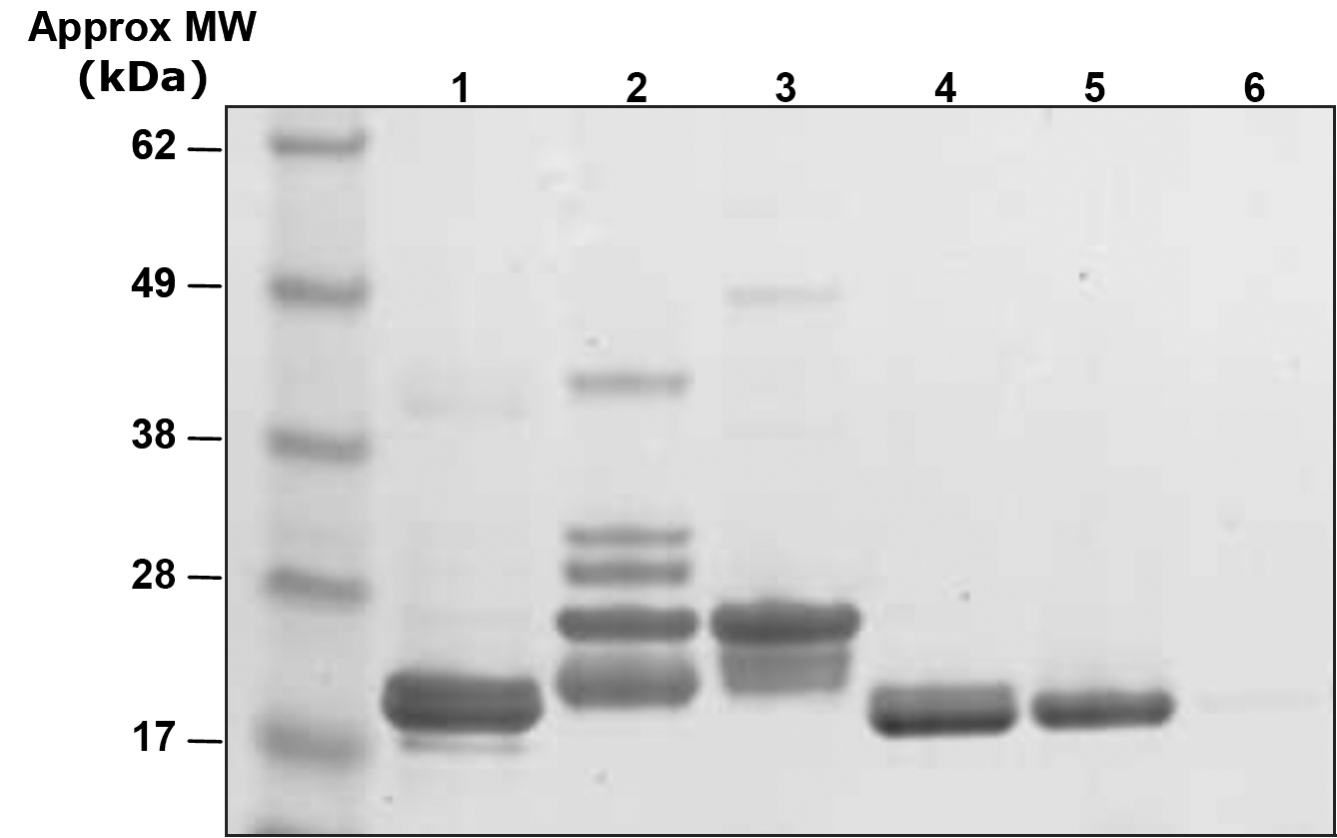
Representative SDS gel electrophoresis results from the water-soluble protein fraction of porcine lens Layer 1.  Lane 1=HMW peak; Lanes 2 and 3=MMW peaks; Lanes 4-6=LMW peaks.  Constituents of the HMW peak correspond to α-crystallins, constituents of MMW peaks to β-crystallins and constituents of the LMW peaks to γ-crystallins.

When analyzed electrophoretically (Figure 3), proteins from the HMW peaks across all lens layers show the double bands at around 19 and 20 kDa that are characteristic of α-crystallin [6]. Fractions from MMW peaks from all layers are dominated by bands between 23 and 32 kDa. Bands around 23–25 kDa are particularly prominent and appear in both MMW peaks; bands around 23 kDa are likely to correspond to the main β-crystallin subunit, βB2-crystallin (formerly βBp-crystallin) [24]. One clear difference between the βH- and βL-crystallin aggregates is the presence in βH-crystallin of polypeptides in the molecular range 28–30 kDa. Bands around 28 kDa are likely to correspond to βB1-crystallin which is specific to βH-crystallin aggregates [24]. All bands decrease in intensity toward the inner layers with higher MW β-crystallin bands disappearing earlier than the 23–25 kDa species that persist in all but the innermost layer. Bands at around 31 kDa are only visible in outermost fractions eluting with the βH-crystallins. The LMW group of proteins was found by SDS gel electrophoresis to consist mainly of γ-crystallins with bands around the 19–21 kDa range. Electrophoresis shows that the main constituents of the water-insoluble fractions are α-crystallin subunits with the presence of some faint bands corresponding to other crystallin classes.

**Figure 4:**
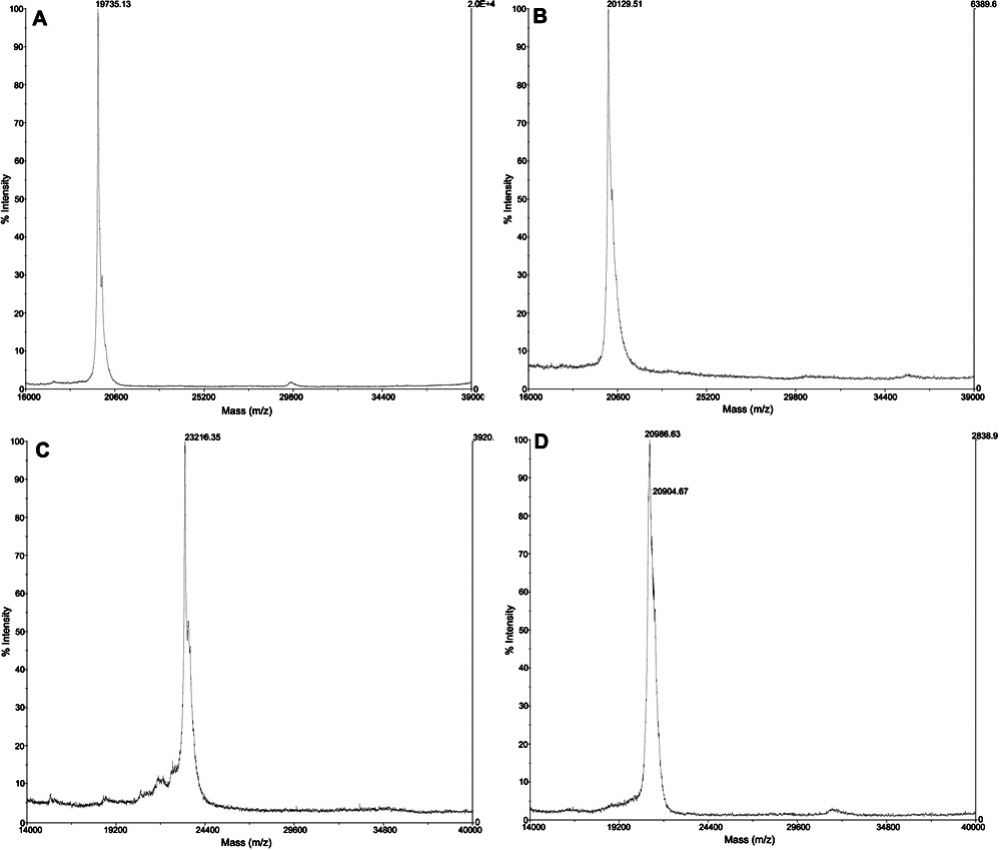
MALDI-TOF mass spectrograms from RP-HPLC fractions of αA-crystallin (**A**); αB-crystallin (**B**); βB2-crystallin (**C**), and a γ-crystallin species (**D**). αA-crystallin=19.735 kDa, αB-crystallin=20.129 kDa, βB2-crystallin=23.216 kDa, a γ-crystallin=20.987 kDa.

RP-HPLC results from the HMW peak yielded two separate peaks the masses of which were found to be 19.735 kDa and 20.129 kDa by MALDI-TOF mass spectrometry (Figure 4A,B). These correspond to αA- and αB-crystallin, respectively. The subunit ratio of αA- to αB-crystallin was 3:1. RP-HPLC results from the larger MMW size-exclusion peak yielded two separate peaks. Only one β-crystallin subunit, with a molecular mass of 23.216 kDa could be identified in each of these peaks (Figure 4C). This appears to correspond to the most abundant gene product of the β-crystallin family, βB2-crystallin. RP-HPLC results from the smaller of the MMW peaks produced one major peak. The mass determined by MALDI-TOF for this peak was also 23.216 kDa, which corresponds to βB2-crystallin. RP-HPLC of pooled LMW fractions produced two main peaks with molecular masses determined as 20.987 kDa and 19.764 kDa (Figure 4D). These masses correspond to γ-crystallin subunits.

## Discussion

The lenses used in this study were from animals that were slaughtered between 5 and 6 months of age and hence unlikely to have undergone any significant age-related alterations or modifications. The protein distribution patterns measured are therefore indicative of the proportions laid down during the course of growth and provide a foundation for further studies that may consider age-related changes. In relation to other species, comparatively few studies have investigated porcine lens crystallin distribution or characterization [17,24-27], in spite of results showing that the porcine lens is a good model for the human lens [17,19,20]. Closeness in sequence homology of αB-crystallin from porcine and human lenses [19] suggests that similar functional processes may operate to maintain transparency in the two species. Immunochemical studies showing cross-reactivity between antibodies against human lenses and porcine lens antigens [20] indicate analogous immune response functions. Similarities in viscoelastic properties between porcine and young human lenses add further support for the use of the porcine lens as a model for the healthy human lens [21].

This study has found that in young porcine lenses, the distributions of the soluble crystallins are relatively constant across most of the lens: α-crystallins represent about 35% of the total soluble protein content, β-crystallins 40%–45%, and γ-crystallins around 12%. In the inner 20%–25% of the lens (taken as a proportion of cumulative protein), there is a slight decrease in the amounts of α- and β-crystallins and an increase in γ-crystallins. The proportion of insoluble protein out of total protein rises continually from the outer to inner lens, reaching a maximum of around 25%. This is higher than the proportions reported by Jobling et al. [17] who found an almost constant insoluble protein level of around 5%. With regard to soluble protein proportions, Jobling et al. [17] found a slightly higher level of HMW (44% in outer lens layers decreasing to 32% in inner layers) than was found in this study. Part of this difference could be accounted for by the higher levels of insoluble protein found in this study and the fact that the HMW proteins reported by Jobling et al. [17] consisted of mixed protein aggregates as well as α-crystallin. The β-crystallin proportions reported by Jobling et al. [17] are comparable to those found in this study but there is a difference with respect to the γ-crystallin patterns. This study found that γ-crystallin constitutes 12% in the outer lens, rising to 18% in the inner lens; the previous study reported up to 30% γ-crystallin in the lens central regions with less that 5% in the outer layers. Comparison with another ungulate shows that some difference in protein proportions exists between porcine and bovine lenses [7,8]. In the latter, the most abundant protein species are α-crystallins, representing around 45% of the total protein over a greater part of the lens [7,8].

In the inner lens layers electrophoresis of soluble proteins showed trace amounts of minor constituents such as a 17 kDa species; these have been observed before in human and bovine lenses [7,28,29] and may be a manifestation of modifications and degradations to the αA- and αB-crystallin chains [10,30,31]. The nomenclature used to describe porcine crystallin subunits has not been consistent. Vidal and Cabezas-Cerrato [26] electrophoretically distinguished 11 significantly different porcine crystallin bands. The authors labeled these bands as α-a, α-b, β-ab, β-c, β-de, β-f, β-g, β-h, β-i, γ-a, and γ-b, and, apart from β-h and β-i, these corresponded closely to bands from gel electrophoresis of bovine proteins [26]. A more conventional classification, used extensively with bovine and human crystallins, has recently been applied to porcine crystallins [27] and has been used in this study.

The molecular masses of the HMW proteins determined using MALDI-TOF (αA-crystallin 19.735 kDa and αB-crystallin 20.129 kDa) agree quite closely with ESI measurements of porcine αA- (19.791 kDa) and αB-crystallins (20.172 kDa) [32]. αA-crystallin has been found to be lens-specific whereas αB-crystallin exists in other tissues [33,34]. Sequence comparison of both bovine αA-crystallin (Swiss-Prot P02470) and bovine αB-crystallin (Swiss-Prot P02510) showed 97.7% and 98.3% homology respectively with their porcine counterparts (Swiss-Prot P02475 and Q7M2W6). Similar findings have been reported previously [19].

β-crystallins encompass several gene products [35-37] and mammalian β-crystallins have been shown to consist of at least seven subunits that can be divided into acidic (βA1–4-crystallin) and basic (βB1–4-crystallin) groups with masses between 22 and 35 kDa [37-39]. βB2-crystallin is the main subunit present in native β-crystallins [24,40]. Unlike the α-crystallins, where the number of bands seen on each gel corresponded to the number of peaks isolated by RP-HPLC, not all of the β-crystallin bands seen on SDS electrophoresis gels could be further characterized. This supports previous findings; in bovine lenses, only the molecular mass of βB2-crystallin was identified [41,42]. This was found to have a molecular mass of 23.216 kDa [41,42] which corresponds to the porcine lens results in this study. If, indeed, this protein is βB2-crystallin, it is found across most of the lens except in the innermost region which is laid down in prenatal life. This supports previous findings for the rat lens in which the βB2-crystallin gene was shown to be expressed in the post-natal lens [43]. The identification of βB2-crystallin in several RP-HPLC fractions of βH- and βL-crystallins suggests that βB2-crystallin may exist in different conformations separated by the RP-HPLC conditions or may aggregate with migration through the column [42]. Sequence analysis for porcine βB1-crystallin (Swiss-Prot Q007T1) shows 94.1% homology with its bovine counterpart (Swiss-Prot P07318). Sequences for other porcine β-crystallins such as βB2-crystallin were not found.

Monomeric γ-crystallins have been found to be composed of as many as 7 subunits with molecular masses around 20 kDa [37]. Another member of the β/γ-crystallin superfamily: monomeric γS-crystallin (formerly βS-crystallin), is believed to be derived from a gene that was separated, during evolution, from the γ-crystallin gene family [44]. In a previous study on fetal human lenses, He et al. [39] were able to identify five separate peaks from γ-crystallin size-exclusion fragments. Kilby et al. [45] confirmed the molecular masses of 3 γ-crystallins from bovine lenses by fast protein ion-exchange chromatography and ESI mass spectrometry. In the present study, the γ-crystallins were partially fractionated into two peaks using RP-HPLC. The major constituent of the larger of these peaks was found to have a molecular mass of 20.987 kDa. These results are comparable to the findings of Vidal and Cabezas-Cerrato [26] who reported masses of between 18.5-19.2 kDa and 19.8-20.3 kDa (from electrophoresis) for the γ-crystallin subunits. No sequence data for any porcine γ-crystallin was found in available databases. However, from the data available for bovine γ-crystallins the species shown in Figure 4D corresponds most closely in mass to γII-crystallin (Swiss-Prot P02526). Further work is required to obtain complete characterization of porcine γ-crystallins.

The results from this study show that in the porcine lens, the proportion of γ-crystallins is the lowest of the three crystallin classes which confirms earlier findings [17,27]. The most abundant protein class, the β-crystallins, constituted only a slightly higher proportion than α-crystallins. In the young human lens the proportions of α- and β-crystallins are around 30% [8]. When taking into account the fact that, in the human lens, the LMW proteins were found to contain significant amounts of β-crystallins [8], the proportions of the soluble crystallin classes in the outer regions of the porcine and young human lens are comparable, which supports the conclusions reached by Jobling et al. [17].

Protein concentrations are linked to the refractive index [18]. Both porcine and bovine lenses have a parabolic refractive index distribution [46,47] that increases to a maximum in the center of the lens and, in both, the level of γ-crystallins also increases in the same direction. This is consistent with the finding that γ-crystallins have the highest refractive increment of all mammalian crystallin classes [48] indicating that this protein class contributes the most to refractive index. It also supports the suggestion that, of all the crystallin classes, γ-crystallins are capable of closest packing [49]. The shape of the refractive index gradient in the porcine lens is similar to its acoustic profile which is also related to protein content [50,51].

The refractive index distribution in the human lens [8,52-55], differs from that found in bovine [46] and porcine [47] lenses; the human lens has a constant refractive index across the nuclear region [8,52-55]. This is supported by the protein concentration profiles [56], water [57] and acoustic gradients [50] found in human lenses. This constant index region may be related not only to protein concentration but also to the fact that across the human lens nucleus there is less variation in LMW protein distribution [8], compared to that found in the porcine lens, as shown in this study and in previous investigations [17]. The low magnitude of refractive index in the nuclear region of the human lens (circa 1.40–1.41) [8,52-55], compared to several other species [55] may also be consistent with a relatively low level of γ-crystallin [8].

While there is a difference in the shapes of the porcine and human refractive index gradients, the magnitude in the center of the porcine lens (1.396 for 633 nm and 1.404 for 532 nm) [47] was found to be comparable to that of the human lens [8,52-55]. An earlier study on lens dispersion had found slightly higher refractive index values in the inner regions of porcine lenses (1.4218–1.4346 from 650 to 440 nm) [58]. The differences between the dispersion study [58] and the study of refractive index on whole lenses [47] may be partially explained by the fact that the former [58] involved lens bisection and this may have caused some evaporation of the lens, which would increase the refractive index.

The relationship between the refractive index and the protein/water proportions [18] does not take into account the fact that water in the lens exists in different states, some bound to protein and some free and therefore mobile [59-61]. Using nuclear magnetic resonance imaging (NMRI), porcine lens nuclei have been found to contain higher proportions of free water than human lens nuclei and porcine lenses were reported to contain greater total water content than human lenses [61]. A higher proportion of total water would suggest less protein and hence a lower magnitude of refractive index in the porcine lens compared to that of the human. This is not confirmed from measurements of the refractive index profile [47], dispersion [58], or acoustic gradients [50]. In addition, total protein contents, found in this study, do not indicate that there is a lower level of protein in the porcine lens compared to the human lens. Without accurate measures of water states and respective proportions, it can be difficult to estimate total water in the lens. Classification of water states requires review [61] and magnetic resonance methods can be misleading if used as a method of determining refractive index or protein concentration in the intact lens.

Water that is mobile has been shown to increase in the human lens, with age and cataract formation [61,62]. It may appear plausible to suggest that increase in free water is correlated with an increased tendency for proteins to move from the soluble to the insoluble phase; species-related differences render this explanation too simplistic [61]. The nuclei of bovine lenses, that had been artificially aged, had no free water, yet the proportion of free water in the human lens nucleus increases with age [61]. The finding of a higher proportion of free water in the porcine compared to human lens nuclei [61] is unlikely to be explained as a result of aging (although no porcine lens ages or weights, from which age could be derived, were provided in the study [61] it is likely that, as the tissue was obtained from an abattoir, the animals were relatively young). The proportion of free water will depend on protein proportions, interactions and conformations and more free water in the nucleus compared to the cortex may have some correlation to the lower amount of cytoskeletal protein in that part of the lens [63]. Why there should be more mobile water in the porcine lens nucleus compared to that of the human is not clear and would require further studies of protein arrangements in the intact lens. Conformational changes across porcine lenses have been measured on tissue sections with Raman spectroscopy [64]. Greater dispersion in vibrations (C-N protein bonds, C-C lipid bonds, C=C stretching, C-N stretching, and CH_2_ bending) were seen along the visual than the equatorial axis. The wide variations did not provide sufficiently accurate information about porcine protein, water, or refractive index gradients that could be used for comparison with the human lens.

In order for the porcine lens to be a suitable model for the human lens, it not only needs to be sufficiently similar to the latter in structural and functional aspects, but the physiologic changes that occur with age and those involved in the process of cataractogenesis in the porcine lens should be analogous to respective alterations in the human lens. No definitive method of normalizing lens aging across species exists, and samples across varying age ranges can be difficult to obtain for comparative studies. An investigation of spectral attenuation and transmission profiles across the visible wavelength range found that the human lens transmits less light than does the porcine lens [65]. The comparison, however, was between 4 month old pig lenses and human lenses aged between 63 and 73 years. A longitudinal study on the in vivo human lens found a mean decrease in transmittance of 0.508%/year for wavelengths between 415 and 550 nm, over a period of 13 years [66]. No comparable investigation of the porcine lens has been found.

A study that examined age-related changes in the proportions of porcine water soluble crystallins, reported a decrease with age in α-, βH-, and βL1-crystallins with a concomitant increase in βL2- and total γ-crystallins although γS-crystallin was found to decrease with age [27]. In the human lens, the proportion of γS-crystallin increases in early post-natal life reaching a constant level from 30 weeks following birth [67]. The predominant decrease in crystallin synthesis with age [27] is consistent with lower protein concentrations and refractive index magnitudes in lens outer layers [47] and with the age-related decrease in the cortical index gradient that has been found in the human lens [53]. The decrease in α- and β-crystallins from the water soluble fractions in the porcine lens can be explained by an increase in protein insolubilisation [27], (also found in the human [8] and bovine lens [68]). This does not, however, account for the age-related changes in the proportion of γ-crystallin [68].

Increasing amounts of insoluble protein extracted from the lens are considered to be an indication of aging and of changes that lead to cataract formation. Protein insolubilization leading to cataractogenesis has been linked to an alteration in Ca^2+^ homeostasis and an overactivation of calpains [69-73]. Porcine lenses with (induced) advanced cortical cataracts were found to have comparable levels of total Ca^2+^/kg lens water [74] to those found in human lenses with advanced age-related cortical cataracts [73]. This suggests that there may be similarities in the calpain activation levels in porcine and human lenses and that the porcine lens may serve as a suitable model for human cataract and for testing calpain inhibitors [74].

The porcine eye has also been used as a model for experimenting with procedures used in cataract surgery [75-77]. Different types of phakic intraocular implants (IOLs) were inserted into porcine eyes to investigate changes in dynamics of the aqueous humor and the causes of secondary cataract that can occur in human eyes following IOL implantation [75]. Effectiveness of ultrasound phakoemulsification [76] and analysis of epithelial cell adhesion to IOLs [77] have also been tested on the porcine eye as a model for its human counterpart.

The protein distribution patterns in the porcine lens lend support to the suggestion that this lens may serve as a suitable model for the human lens. Further studies are needed on the structure/function relationships between the proteins of the lens and its optical function in the two species to investigate the similarities and the differences with growth, aging and cataractogenesis.
